# Immunotherapy in Cervical and Endometrial Cancer: Current Landscape and Future Directions

**DOI:** 10.3390/life14030344

**Published:** 2024-03-06

**Authors:** Dimitrios Stefanoudakis, Evaggelia Karopoulou, Alkis Matsas, Georgia Anna Katsampoula, Ermioni Tsarna, Eleni Stamoula, Panagiotis Christopoulos

**Affiliations:** 1 Second Department of Obstetrics and Gynecology, Aretaieion University Hospital, Medical School, National and Kapodistrian University of Athens, 76 Vasilissis Sofias Avenue, 11528 Athens, Greece; stefanoudak@med.uoa.gr (D.S.); amatsas@med.uoa.gr (A.M.);; 2 First Department of Propaedeutic Surgery, Hippocration Hospital, Athens Medical School, National and Kapodistrian University, 11528 Athens, Greece; 3 Department of Clinical Pharmacology, School of Medicine, Aristotle University of Thessaloniki, University Campus Aristotle University of Thessaloniki, 54124 Thessaloniki, Greece

**Keywords:** immunotherapy, cervical cancer, endometrial cancer, immune checkpoint inhibitors, cancer vaccines, adoptive cell therapies, treatment resistance, biomarkers

## Abstract

Gynecological cancers pose a significant burden on women’s health worldwide, necessitating innovative treatment approaches. Immunotherapy has emerged as a promising strategy, harnessing the body’s immune system to combat cancer. This review aims to provide a comprehensive overview of the current landscape and future directions of immunotherapy in cervical and endometrial cancer. Methods: A thorough literature search was conducted to identify relevant studies and clinical trials. The main methods and treatments employed in immunotherapy for cervical and endometrial cancer, including immune checkpoint inhibitors, cancer vaccines, and adoptive cell therapies, are briefly described. Results: Immune checkpoint inhibitors, such as anti-PD-1/PD-L1 antibodies, have shown remarkable clinical efficacy in certain gynecological malignancies, particularly in advanced or recurrent cases. Additionally, ongoing research on cancer vaccines and adoptive cell therapies holds promise for personalized and targeted treatment options.

## 1. Introduction

Gynecological cancers are prevalent among women worldwide, affecting their physical, psychological, and social well-being. These cancers disrupt reproductive organ function, impacting sexual life, self-esteem, and fitness. Treatment side effects further affect the quality of life. Clinical management is multifaceted, considering patient age, performance status, and comorbidities. In the US, approximately 115,000 gynecological cancer cases with 34,000 deaths were reported in 2023. More specifically, cervical cancer affected 13,960 women, with 4310 deaths, with socioeconomics and ethnicity influencing screening and survival rates. Endometrial cancer was mostly diagnosed in women aged 55–64 years, with 66,200 reported cases in 2023 [1,2]. Additionally, in Europe, it is estimated that there have been around 61,100 new cases of cervical cancer and 25,800 related deaths [3]. Early detection remains a challenge in all gynecological cancers, impacting survival rates [1,2]. The traditional treatment of gynecological cancers typically involves a combination of chemotherapy and surgery. For advanced-stage cases, debulking surgery is recommended to remove as much tumor tissue as possible, leading to better treatment outcomes [4]. As an alternative, immunotherapy has surfaced as a promising pathway in the treatment of gynecological cancers, offering patients the potential for enhanced results and prolonged survival.

## 2. Principles of Immunotherapy

The body’s defense against pathogens comprises two main components: innate immunity and adaptive immunity. Innate immunity serves as the initial line of defense, employing physical and chemical barriers such as the skin and mucosal surfaces, along with cellular responses. In contrast, adaptive immunity is antigen-specific and memory-based, involving B and T lymphocytes. T-cell activation relies on T-cell receptors (TCRs) recognizing antigens presented by MHC class I or II molecules, with CD8+ T cells responding to endogenous antigens and CD4+ T cells to exogenous antigens. Co-stimulation through molecules like CD28 is crucial for T-cell activation. Cytokines play a vital role in shaping T-cell differentiation and function. B-cell activation occurs when antigens crosslink membrane immunoglobulin receptors, with T-cell assistance required. Memory B cells retain antigen information, and immunologic memory involves the clonal expansion of antigen-specific lymphocytes, leading to a quicker and more robust response upon re-exposure to the same antigen. Together, innate and adaptive immunity provide a comprehensive defense strategy against a wide range of pathogens [5].

In recent years, the immune system’s role in cancer control has gained recognition, with a focus on both adaptive and innate immune responses. Tumor-infiltrating lymphocytes, particularly CD8+ cytotoxic T cells, and the balance between CD8+ and CD4+/forkhead box P3+ regulatory T cells in the tumor microenvironment have emerged as critical factors. Dysfunctional immune interactions and evasion mechanisms often impede immune responses to cancer. Immunotherapy, including immune checkpoint inhibitors, cancer vaccines, and adoptive T-cell therapy, holds promise for enhancing antitumor immune responses and improving cancer treatment [6]. Cancer immunotherapy, with its roots dating back to the late 19th century and milestones including FDA-approved treatments like interferon-α and interleukin-2, has made significant progress. The discovery of tumor-associated antigens, the development of peptide-based cancer vaccines, and the use of toll-like receptor ligands as adjuvants have been pivotal. Immune checkpoint inhibitors, specifically anti-PD-1, anti PD-L1, and anti-CTLA4 antibodies, have transformed cancer treatment. Dendritic cell-based therapies and monoclonal antibodies targeting tumor survival molecules have shown promise. Ongoing research aims to identify new antigens, improve vaccine efficacy, and overcome immunosuppression, offering hope for advanced cancer treatment [7].

The microbiome, crucial for human health, particularly influences gynecologic cancers. Dysbiosis in gastrointestinal and urogenital tracts, influenced by genetics, lifestyle, and environment, correlates with cancer development. Vaginal dysbiosis, including decreased Lactobacillus and increased anaerobic bacteria, is associated with higher HPV risk and cervical neoplasia. Microbiota alterations during cancer therapy impact treatment response; chemotherapy-induced dysbiosis exacerbates gastrointestinal toxicity. The gut microbiota modulates immune responses to cancer therapy, affecting efficacy and toxicity. Antibiotic use prior to immunotherapy detrimentally affects outcomes in gynecologic cancers, highlighting the microbiome’s role in treatment response and suggesting potential therapeutic strategies through microbiota modulation [8,9].

Recent research has highlighted the correlation between improved survival and the presence of tumor-infiltrating effector lymphocytes in ovarian cancer patients, emphasizing the importance of immune surveillance in the disease. Immunotherapy efforts have explored vaccination trials involving whole tumor cells and tumor cells loaded onto dendritic cells (DCs) to stimulate immune responses against a wide range of ovarian carcinoma-specific antigens. Clinical trials using DC-enriched peripheral blood mononuclear cells loaded with HER-2/neu-GM-CSF have shown some clinical activity, but their impact on survival, similar to sipuleucel-T in prostate cancer, is yet to be determined for ovarian cancer patients [10,11,12,13].

Cancer immunotherapy faces challenges related to dysfunctional antitumor T cells and an immunosuppressive tumor microenvironment (TME). Immune checkpoint inhibitors have shown success in specific cancers, but overall responses vary. Combining chemotherapy with immunotherapy has shown promise, with certain drugs like taxanes enhancing immune responses. Radiation therapy (RT) triggers immune responses but also recruits immunosuppressive cells. Combining RT with immunotherapy aims to harness its benefits. Additionally, cancer vaccines combined with immune checkpoint inhibitors show potential for improving outcomes. These combinations are under exploration in clinical trials, offering promising avenues for a wide range of diseases, either as standalone treatments or in combination with other therapies [14,15]. Immunotherapy encompasses a wide array of strategies, including cytokine therapy, T-cell engineering, immune checkpoint inhibitors, monoclonal antibodies, and stem cell transplantation. These diverse approaches offer versatile tools for combating various diseases, from cancer to infectious diseases, by either directly modifying immune cells or unleashing the body’s immune response through various mechanisms [15,16].

## 3. Immunotherapeutic Approaches

### 3.1. Immune Checkpoint Inhibitors

**Figure 1:**
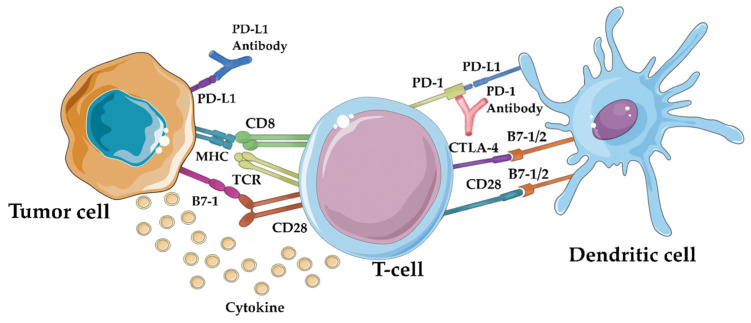
Immune checkpoint inhibitors’ mechanism of action: by blocking the PD-1/PD-L1 interaction, immune checkpoint inhibitors like anti-PD-1 and anti-PD-L1 antibodies can “release the brakes” on the immune system, enabling it to recognize and attack tumor cells more effectively [20].

Immune checkpoint inhibitors (ICIs) like PD-1 and CTLA-4 inhibitors have revolutionized cancer treatment by targeting receptors that modulate the immune response. They block inhibitory signals, allowing immune cells to mount a more effective response against cancer cells. These ICIs operate through distinct mechanisms: CTLA-4 inhibitors boost tumor-specific T-cell activation and proliferation by promoting CD28-mediated co-stimulation, while PD-1/PD-L1 inhibitors restore the function of tumor-infiltrating T cells by reversing negative signaling [17]. Figure 1 shows schematically the above. At present, immune checkpoint inhibitors represent the most successful immunotherapeutic approach due to their peculiar ability to target lymphocyte receptors, as opposed to current targeted therapy, such as bevacizumab, trastuzumab, and cetuximab, that acts directly on the tumor cells [18]. FDA-approved antibodies targeting these checkpoints—ipilimumab, pembrolizumab, nivolumab, cemiplimab, avelumab, atezolizumab, and durvalumab—have shown clinical benefits in various cancers. While biomarkers like tumor mutational burden and PD-L1 staining are being explored for treatment response prediction, ICIs have also gained approval for gynecologic malignancies, expanding their impact in the field of immunotherapy [19].

### 3.2. Cancer Vaccines

Cancer vaccines are a promising approach in cancer immunotherapy, aiming to trigger specific and long-lasting immune responses against tumor antigens (TAs), which include mutational antigens derived from mutated self-proteins and tumor-associated antigens (TAAs), and non-mutated proteins overexpressed in cancer cells. While only one therapeutic cancer vaccine has gained approval for human use, research is ongoing to enhance DNA vaccine efficacy through various strategies, such as combining them with immunostimulatory cytokines, immune checkpoint blockade therapies, low-dose chemotherapy, endocrine therapy, and radiotherapy. These vaccines are being explored in clinical trials for safety and immunological responses against various cancer types. Understanding the immunological characteristics of cancers and complexities of the tumor microenvironment and selecting appropriate antigens are crucial aspects of vaccine development. The field encompasses various vaccine approaches, including peptide- and protein-based vaccines, cellular vaccines, and genetic vaccines, with advancements in shared tumor-associated antigens (TAAs) and personalized neoantigens, novel vaccine platforms, and adjuvants showing promise for more effective and personalized cancer treatments in the future. Overcoming therapy resistance involves tailored combinations addressing both tumor and microenvironment factors to improve outcomes in cancer immunotherapy [21,22,23,24].

### 3.3. Adoptive Cell Therapies 

**Figure 2:**
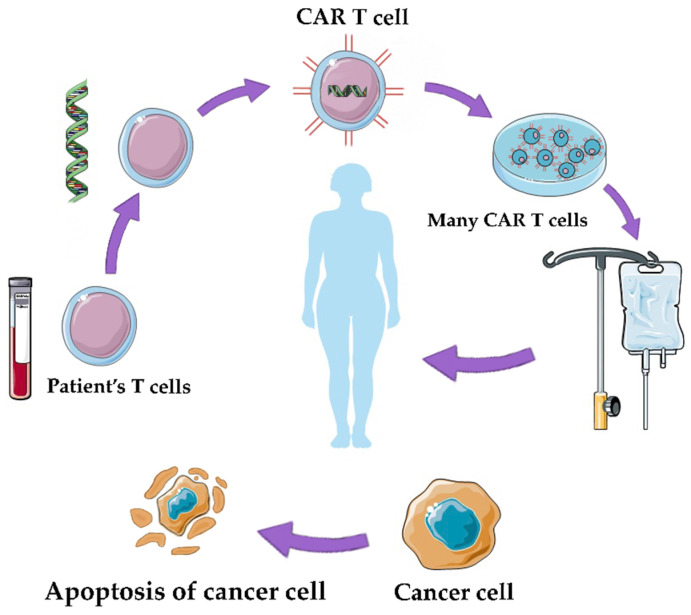
CAR-T therapy: the therapy involves the genetic modification of a patient’s own T cells to enhance their ability to target and destroy cancer cells [20].

Adoptive cell therapy (ACT) is a promising approach in cancer treatment, utilizing genetically engineered T cells to target and eliminate cancer cells. Two main forms of ACT, TCR-T therapy and CAR-T therapy, have shown efficacy but can lead to severe side effects. Strategies to mitigate these effects include incorporating safety switches, reducing receptor affinity, and using logic gate CARs to enhance specificity. A major challenge in ACT is identifying high-specificity neoantigens that can reduce the need for complex safety controls. Researchers are using methods like whole-exome sequencing (WES) combined with mass spectrometry (MS) to predict immunogenic neoantigens by analyzing tumor-specific mutations and identifying proteins associated with HLA-1. Notable neoantigens like EGFRvIII, KRAS mutant, MYD88 mutant, IDH1 mutant, mutant p53, and MUC1 alterations have demonstrated potential in cancer immunotherapy. These neoantigens offer insights into enhancing cancer treatment approaches, but ongoing research is needed to streamline neoantigen discovery across various cancer types and patients. ACT offers innovative strategies for combating solid tumor malignancies, including tumor-infiltrating lymphocytes (TILs) and genetically engineered T cells like TCR gene therapy and CAR T-cell therapy. Challenges include managing toxicities and optimizing ACT for a broader range of cancers, especially solid tumors, through advances in genetic engineering techniques like CRISPR to enhance T-cell function and overcome immunosuppressive tumor microenvironments [25,26,27]. Figure 2 presents the method of production of CAR T cells.

### 3.4. Combination Approaches 

Enhancing the effectiveness of cancer immunotherapy involves a two-phase strategy for advanced tumors resistant to single-agent immune checkpoint inhibitors (ICIs). The first phase aims to reduce the tumor burden through treatments such as surgery, chemotherapy, or radiotherapy, which can also alleviate immunosuppressive pathways. In the second phase, therapeutic vaccines, often personalized neoantigens, are administered, typically in the form of peptides, RNA, or DNA. These vaccines are combined with chemotherapy, ICIs, or other immunomodulatory treatments in order to reinvigorate exhausted T cells within the tumor microenvironment. Timing is crucial, and combining therapeutic vaccines with ICIs has shown promise, especially in HPV-associated cancers. Additionally, utilizing immunostimulatory agents, targeting immunosuppressive factors in the tumor microenvironment, and combining therapeutic vaccines with adoptive cell therapy represent emerging approaches for maximizing the clinical impact of cancer immunotherapy [23]. Combination strategies leverage the immune system’s role in cancer progression, aiming to enhance tumor-specific immune responses, reduce tumor burden, and improve treatment outcomes through the judicious use of surgery, chemotherapy, radiation therapy, and immunomodulatory treatments, both preclinically and in clinical trials [28].

## 4. Cervical Cancer

Cervical cancer is a global health concern, ranking as the fourth most common cancer among women worldwide, with significant regional variations and a notable impact on eastern, western, central, and southern Africa [29]. Since it is closely linked to HPV infections, HPV vaccines are considered a critical preventive tool. Despite progress in vaccination and screening, challenges persist, with suboptimal vaccine coverage and screening rates in the United States. Immunotherapy holds promise in cervical cancer treatment, targeting HPV-related viral proteins. ISA-101, in combination with nivolumab, has shown a promising response rate. Since traditional treatment options such as surgery, radiation therapy, and chemotherapy have poor outcomes for advanced-stage disease, alternative therapies like bevacizumab have emerged as an intriguing field of exploration. Cervical cancer screening primarily relies on the Pap test and HPV testing, and treatment availability varies by resource, with limited access to radiation therapy and palliative care in low-resource settings. Addressing disparities in vaccination, screening, treatment, and palliative care is crucial for effectively combating cervical cancer, especially in resource-constrained regions [18,29,30,31].

### 4.1. Immune Checkpoint Inhibitors

Advancements in cervical cancer management encompass several key areas. These include improving screening through trials evaluating HPV self-collection kits for high-risk women, exploring conservative treatments for early-stage cervical cancer to preserve fertility, and the growing prominence of immunotherapy as a second-line treatment for non-responsive patients. Pembrolizumab, approved for cervical cancer patients with a PD-L1 mutation, has shown promise, with ongoing trials examining combination immunotherapies and vaccine approaches targeting HPV-related cancers. Checkpoint inhibitors combined with radiation therapy and innovative strategies like adoptive cell therapy using autologous tumor-infiltrating lymphocytes are also in development. However, vigilance is required due to the unique side effects of immunotherapy. These ongoing research efforts hold the potential to shape the future of cervical cancer management, providing hope for patients with limited treatment options. Immune checkpoint inhibitors such as pembrolizumab and nivolumab are being tested with varying response rates, and their efficacy is linked to PD-L1 expression. Additionally, ipilimumab and other checkpoint inhibitors are being explored, necessitating further research to fully harness the potential of immunotherapy in cervical cancer treatment [17,18,32].

Ipilimumab, an immunotherapy drug, was studied in a phase 1/2 trial involving women with metastatic or recurrent HPV-related cervical carcinoma, with limited efficacy observed and notable toxicities, highlighting the need for alternative treatments [33]. Pembrolizumab, a PD-1 receptor-blocking monoclonal antibody, demonstrated favorable outcomes in advanced cervical cancer patients, with an objective response rate (ORR) of 12.2% in the KEYNOTE-158 trial [34] and 17% in the KEYNOTE-028 trial [35], leading to FDA approval for relapsed or metastatic cervical cancer with PD-L1 expression [36,37]. Nivolumab, another PD-1 receptor-targeting antibody, exhibited substantial antitumor activity in recurrent or metastatic cervical cancer, with a 26.3% ORR in the CheckMate 358 trial [38] and positive results in the NRG-GY002 trial [39,40,41]. However, a study combining bevacizumab and atezolizumab did not enhance the objective response rate in advanced cervical cancer [42]. Ongoing trials are evaluating the potential benefits of durvalumab as an adjuvant therapy following chemoradiotherapy [43,44,45,46]. Additionally, CTLA-4 blockade with ipilimumab is being explored in cervical cancer treatment [47,48]. Preliminary results from the ongoing phase I/II trial of balstilimab (anti-PD-1) as monotherapy or in combination with zalifrelimab (anti-CTLA-4) in patients with metastatic or locally advanced cervical cancer were also presented [49,50]. The ORR was 14% for balstilimab monotherapy and 22% for the combination regimen. Additionally, the median duration of response (DOR) was 15.4 months for balstilimab monotherapy and not reached for the combination treatment. Treatment was well tolerated, with manageable immune-related adverse effects occurring in approximately 30–35% of patients. These findings highlight the potential of dual PD-1/CTLA-4 blockade as a promising therapeutic approach for cervical cancer patients. Several ongoing phase II and III clinical trials are investigating the efficacy and safety of immune checkpoint inhibitors (ICIs) in advanced cervical cancer. The Gynecologic Oncology Group (GOG)-3028 (RaPiDs) trial is evaluating balstilimab monotherapy and balstilimab with zalifrelimab combination therapy in patients who relapsed or progressed after first-line platinum-based chemotherapy. In the first-line setting, the addition of atezolizumab to platinum-based chemotherapy, paclitaxel, and bevacizumab is being assessed in a phase III trial (BEATcc Study) [51] and the efficacy and safety of pembrolizumab in combination with platinum-based chemotherapy (with or without bevacizumab) is being investigated in another phase III trial (KEYNOTE-826) [52] for persistent, recurrent, or metastatic cervical cancer. Additionally, phase II trials are exploring pembrolizumab as an add-on to standard-of-care pelvic cisplatin-based chemoradiation for locally advanced cervical cancer [53] and evaluating pembrolizumab with concurrent chemoradiotherapy in patients with high-risk, locally advanced cervical cancer who have not undergone systemic therapy, immunotherapy, definitive surgery, or radiation (KEYNOTE-A18; ENGOT-cx11) [54]. Moreover, a multicenter phase 2 trial in Japanese patients indicated positive outcomes with nivolumab in cervical and corpus cancers [55]. These trials collectively suggest that immunotherapy, particularly PD-1 inhibitors like pembrolizumab and nivolumab, holds promise as a treatment option for advanced cervical cancer, especially in cases resistant to standard therapies, but challenges remain in optimizing patient selection and response rates.

### 4.2. Cancer Vaccines 

Several studies have explored immunotherapy approaches for cervical cancer. In one study involving 29 patients with early-stage cervical cancer, a recombinant vaccinia virus vaccine called TA-HPV was evaluated. The vaccine induced immune responses, including antibodies specific to HPV-16 and HPV-18, and cytotoxic T-lymphocyte responses in some patients, but HLA loss in tumor biopsies suggested immune evasion by tumors [56]. In another phase I trial, dendritic cell (DC) vaccination was assessed in 14 patients with early-stage cervical cancer, showing safety and increased immune responses against HPV-16/18 E7 and KLH antigens [57]. A separate study involving 32 heavily pretreated advanced cervical cancer patients used pre-immature dendritic cells (PIDCs) pulsed with HPV16 E6 or E7 peptides and observed immunological responses in 61% of patients, although clinical benefits were limited [58]. Lastly, a phase I trial of a peptide-based HPV therapeutic vaccine for CIN2/3 demonstrated safety, efficacy in regressing CIN2/3 lesions, and the induction of T-cell responses, highlighting its potential for cervical intraepithelial neoplasia treatment [59].

Numerous clinical studies have explored various immunotherapy approaches for cervical cancer and pre-malignant cervical conditions. In one study, a peptide-based HPV16 therapeutic vaccine was tested in 51 women with low-grade pre-malignant cervical disorders, demonstrating safety, immune response enhancement, and clinical regression of abnormalities in some cases [60]. Another phase 2 clinical study involving patients with refractory/persistent uterine cervical and ovarian cancer utilized a cocktail vaccination of cancer-derived multiple-epitope peptides, showing promising disease control rates and overall survival, particularly in patients with specific clinical characteristics [61]. In a phase 1 study, an HPV16-specific immunotherapy for women with cervical intraepithelial neoplasia (CIN) induced significant humoral and cell-mediated immune responses, with reductions in HPV viral load [62]. In another phase I/II trial, the HspE7 vaccine was evaluated in patients with CIN3 or CIN2-3, achieving complete or partial pathologic responses and demonstrating safety [63]. Additionally, a study investigating the GX-188E therapeutic DNA vaccine in patients with CIN3 showed remarkable clinical responses, complete lesion clearance, and HPV clearance in a significant proportion of patients [64]. The MVA E2 therapeutic vaccine exhibited effectiveness in clearing high-grade cervical lesions and reducing recurrence rates in patients with HPV infections. Furthermore, TG4001 immunotherapy demonstrated promising clinical efficacy, with some patients achieving complete lesion regression and HPV clearance [65]. In another study, the MVA E2 recombinant virus vaccine successfully induced complete regression of low-grade and high-grade cervical lesions, significantly reducing recurrence rates [66]. A combination therapy involving rhAd-p53 and chemotherapy showed promise in reducing tumor size and cancer-related markers in locally advanced cervical cancer, with a notable improvement in the combined treatment group [67]. In a phase I/II trial, an HPV type 16 E7 protein-based vaccine combined with an adjuvant demonstrated safety and immunogenicity in women with cervical intraepithelial neoplasia (CIN). The vaccine induced CD8+ T-cell responses, sustained up to two years, and weak-to-moderate humoral responses, including anti-E7 antibodies [68]. In a separate phase II study, a vaccine targeting high-grade cervical intraepithelial neoplasia (CIN II and CIN III) displayed immunogenicity in over half of the patients, with some achieving complete responses and HPV clearance [69]. Another study explored TG4001 immunotherapy in patients with high-grade cervical intraepithelial neoplasia (CIN 2/3) and HPV 16 infection, showing clinical and virological responses, including complete lesion regression and HPV clearance, supporting the concept of HPV-targeted immunotherapy [70]. Moreover, ADXS11-001 immunotherapy targeting HPV-E7 in Indian women with recurrent cervical cancer demonstrated a disease control rate of 38% and promising survival rates, supporting its role as an alternative treatment option with lower toxicity compared to conventional chemotherapy [71].

Lastly, topical immunotherapy can be used as a combination with therapeutic vaccines. Imiquimod, an immunomodulatory drug, shows promising effectiveness in treating HPV-related neoplasms of the female lower genital tract. Acting as a TLR7/8 agonist, it stimulates innate immunity by inducing interferon production and inflammatory molecule release, leading to antigen presentation, T-helper lymphocyte activation, and immune microenvironment polarization. Clinical evidence highlights its efficacy in treating VHSIL, VaIN, and CIN, with high response rates observed, including sustained remissions and HPV clearance. The immune microenvironment, particularly infiltration by specific immune cells, serves as a predictor of treatment response. Despite well-tolerated side effects, including transient local and systemic reactions, imiquimod’s ability to induce apoptosis in HPV-positive cells and inhibit proliferation enhances its therapeutic potential. Emerging formulations like nanoencapsulated imiquimod show promise in pre-clinical studies. Further research is needed to translate these findings into clinical practice and advance personalized medicine for HPV-related neoplasms [72].

### 4.3. Adoptive Cell Therapies 

In a clinical trial involving 17 patients with advanced metastatic cancer, TCR-transduced CD4+ T-cell therapy was evaluated. The study found no dose-limiting toxicities during dose escalation, enabling the highest cell dose for nine patients. In the low-dose phase, a cervical cancer patient achieved an ongoing complete response, and three patients in the high-dose group showed objective responses. Some patients experienced transient grade 3 toxicities and high fever, primarily due to chemotherapy and IL-2. Elevated levels of IL-6 and IL-10 were detected in the blood after cell infusion. Genetically modified T cells persisted in peripheral blood, particularly in high-dose recipients, although this did not consistently correlate with clinical responses. Some patients developed resistance to T-cell therapy, possibly due to antigen loss or presentation pathway dysfunction [73]. In a separate study from 2012 to 2014, nine women with metastatic cervical cancer received TILs targeting HPV E6 and E7 oncoproteins. Three patients achieved objective tumor responses, with two experiencing complete remissions lasting 22 and 15 months, even in cases of prior multiple chemotherapy regimens and widespread metastases. TIL infusion was well tolerated, with no acute toxicities or autoimmune adverse events reported. The study also noted a correlation between HPV reactivity in TILs and clinical response, with responding patients exhibiting prolonged repopulation of HPV-reactive T cells in peripheral blood, emphasizing the potential of immunotherapy in managing metastatic cervical cancer [74].

### 4.4. Combination Approaches

A study evaluated the effectiveness of combining immune therapy with chemotherapy in treating cervical cancer by assessing immune function and recurrence rates. Initially, both experimental and control groups showed no significant differences in T-lymphocyte subsets. However, post-treatment, the experimental group exhibited significantly higher CD3+ CD4+ and CD16+ CD56+ cell levels, indicating improved immune response, while CD3+ CD8+ levels remained unchanged. Notably, the CD4+ CD25+ regulatory T-cell ratio decreased significantly in the experimental group, but increased in the control group after treatment. Examination of perforin, GraB, and CD107a expression in peripheral blood mononuclear cells (PBMCs) revealed decreased levels in the control group post-treatment, contrasting with the significant increases observed in the experimental group. Over a three-year follow-up period, the experimental group had lower recurrence rates (9 cases) compared to the control group (18 cases), with statistically significant differences. Additionally, the cumulative survival rate in the experimental group (80%) significantly outperformed that of the control group (56.41%). In conclusion, this study affirms the effectiveness of biological immune treatment, particularly when combined with chemotherapy, in enhancing immune function and reducing cervical cancer recurrence rates, supporting its clinical applicability in cervical cancer therapy [75].

The abovementioned studies collectively highlight the potential of immunotherapeutic approaches in treating cervical cancer and pre-malignant conditions, offering hope for improved clinical outcomes and reduced toxicity.

## 5. Endometrial Cancer

Endometrial cancer is a prevalent gynecological malignancy, with risk factors linked to estrogen exposure, obesity, and various health conditions. Its most common presentation is post-menopausal bleeding. While routine screening is not widely recommended, women over 65 should be aware of the risks, especially those with Lynch syndrome who may benefit from annual endometrial biopsies starting at 35. Prevention involves managing risk factors and considering progesterone supplementation in hormone therapy. The primary treatment is surgical, involving total hysterectomy and salpingo-oophorectomy, currently often with the detection of sentinel lymph nodes, possibly with radiation and chemotherapy, and prognosis is dependent on disease stage and histology. The tumor microenvironment plays a significant role in endometrial cancer progression, involving immune cells and stromal cells with both pro and antitumorigenic functions. Hormones like estrogen also impact the cancer microenvironment. The type and stage of endometrial cancer guide treatment decisions, including surgery, radiation, and systemic therapies like chemotherapy. Complex atypical endometrial hyperplasia can be treated with hysterectomy, while low-risk cases may opt for non-surgical treatments. Overall, understanding the complexities of endometrial cancer risk factors, diagnosis, treatment, and the tumor microenvironment is crucial for effective management [76,77,78]. Ongoing trials are investigating ICI combinations and improved biomarker selection to enhance ICI efficacy in EC.

### 5.1. Immune Checkpoint Inhibitors

In the KEYNOTE-028 study, pembrolizumab showed durable antitumor activity in PD-L1-positive advanced endometrial cancer patients, with a 13% objective response rate (ORR) and manageable safety. In a study of 41 patients with mismatch repair-deficient tumors, pembrolizumab demonstrated substantial efficacy, with immune-related response rates of 40% in colorectal and 71% in non-colorectal cancers, indicating disease control and prolonged survival, particularly in patients not associated with Lynch syndrome [79]. In contrast, mismatch repair-proficient colorectal cancers did not respond to pembrolizumab. Genomic analysis revealed higher mutational loads and potential neoantigens in mismatch repair-deficient tumors, along with elevated CD8-positive lymphoid cell density and PD-L1 expression in responsive tumors [80]. The KEYNOTE-158 study supported pembrolizumab as a valuable treatment option for previously treated MSI-H/dMMR advanced non-colorectal cancer patients [81]. The GARNET trial highlighted the potential of dostarlimab in dMMR endometrial cancer. Its antitumor activity seems to be significant, with a 43.5% response rate in dMMR/MSI-H patients and 14.1% in MMRp/MSS patients, along with manageable side effects [82]. An avelumab study stratified patients based on mismatch repair status, demonstrating a 26.7% ORR in MMRD patients and highlighting the potential of IHC for patient selection [83]. A separate phase II study investigated avelumab in patients with recurrent endometrial cancer, stratified by mismatch repair status. The study showed a 26.7% overall response rate and 40% progression-free survival at 6 months in the deficient mismatch repair cohort, identifying responders through cost-effective IHC. Some non-responders in this group had JAK1 or B2M mutations, highlighting avelumab’s potential in MMRD endometrial cancer and the importance of IHC for patient selection [84]. In the PHAEDRA trial, durvalumab exhibited significant clinical activity in the dMMR cohort of advanced endometrial cancer patients, with a 47% ORR, while its efficacy in pMMR cases appeared limited [85]. A study investigated the efficacy of pembrolizumab, a PD-1 blockade therapy, in mismatch repair-deficient cancers, including those with Lynch syndrome. The results from 86 patients who had progressive disease after prior therapy showed objective radiographic responses in 53% of patients, with disease control in 77%. Notably, neither median progression-free survival (PFS) nor overall survival (OS) had been reached at the study cutoff, demonstrating the potential of PD-1 blockade across various tumor types, regardless of Lynch syndrome status [86]. Another phase II study found promising responses with pembrolizumab in MMR-deficient endometrial cancer, suggesting a new standard of care for immunotherapy. Additionally, the research highlighted the impact of secondary cytoreductive surgery outcomes on survival in endometrial cancer patients and the comparison of cabozantinib and weekly paclitaxel in recurrent ovarian cancer, showing no significant PFS improvement with cabozantinib and increased gastrointestinal toxicities. These studies offer insights into the treatment options and outcomes for gynecological cancers [87]. Furthermore, in a phase 3 trial, dostarlimab combined with chemotherapy improved progression-free survival in advanced endometrial cancer patients, particularly those with dMMR-MSI-H tumors, with manageable side effects [88]. Lastly, a multicenter phase 2 trial involving Japanese patients underscored the potential of immunotherapy in both endometrial and cervical cancer. Specifically, nivolumab demonstrated promising results in uterine cervical cancer and uterine corpus cancer but limited efficacy in soft tissue sarcoma (STS). The study included 63 cervical cancer, 23 uterine corpus cancer, and 21 STS patients, with overall response rates (ORR) of 25% for cervical cancer, 23% for corpus cancer, and 0% for STS. Disease control rates (DCR) were 75%, 68%, and 48%, respectively. Nivolumab led to tumor size reduction in responsive cervical and corpus cancer patients, while some STS patients achieved disease stability. Subgroup analyses highlighted the influence of PD-L1 status on response in cervical cancer, whereas corpus cancer responses were less influenced by PD-L1 status [55].

### 5.2. Combination Approaches

In a phase 2 study with 77 patients with persistent or recurrent endometrial cancer, treatment with durvalumab alone or in combination with tremelimumab resulted in modest efficacy. In the durvalumab-only arm, the objective response rate (ORR) was 10.8%, with more durable responses in those who achieved complete responses. The combination arm had an ORR of 5.3%, with all responders having complete responses, and the duration of response was promising. Safety profiles revealed immune-mediated adverse events, more frequently in the combination arm. Responses were predominantly seen in patients with deficient mismatch repair (dMMR) tumors, indicating potential benefits in this subgroup but limited efficacy overall [89]. In another study, the combination of epacadostat and pembrolizumab showed promising antitumor activity and manageable safety profiles in advanced solid tumors across various types. The combination was well tolerated, with promising responses, making it a potential treatment option for advanced solid tumors [90]. Lastly, lenvatinib plus pembrolizumab demonstrated meaningful clinical responses in advanced endometrial cancer patients, with a 38.9% overall response rate, a median duration of response of 21.2 months, and manageable but notable treatment-related adverse events, although serious adverse events led to patient discontinuations and treatment-related deaths, suggesting potential clinical benefits but also the importance of careful monitoring and management [91].

## 6. Conclusions

Immunotherapy has emerged as a promising avenue for the treatment of gynecological cancers, providing patients with improved outcomes and extended survival. Nevertheless, several hurdles still need to be addressed in this field. Identifying reliable predictive biomarkers is crucial for selecting patients who are most likely to benefit from immunotherapy. Additionally, understanding and overcoming resistance mechanisms that some patients develop during treatment is essential. Combining immunotherapies with traditional treatment modalities like chemotherapy and radiation therapy may enhance overall treatment efficacy. Moreover, ongoing research should focus on refining immunotherapeutic approaches and developing innovative strategies to boost response rates and prolong response durations. With these efforts and continued clinical trials, immunotherapy has the potential to revolutionize the landscape of gynecological cancer treatment, offering new hope and improved outcomes for patients in the future.

## Data Availability

The data used in this study are presented within the manuscript.
